# Evaluation of nutrients in effluent from wastewater treatment lagoons discharging to surface water in the United States

**DOI:** 10.2166/wst.2026.261

**Published:** 2026-04-18

**Authors:** Ziya Sing-guang Jang, Denis Ruto, Pablo K. Cornejo, Kevin D. Orner, Harold Leverenz

**Affiliations:** a Department of Civil and Environmental Engineering, University of California, Davis, CA, USA; b Department of Civil and Environmental Engineering, West Virginia University, Morgantown, WV, USA; c Department of Civil and Environmental Engineering, California State University, Chico, CA, USA

**Keywords:** ammonia loading, nutrient removal, technology upgrades, temperature effects, waste stabilization ponds

## Abstract

Wastewater treatment lagoons (WTLs) are one of the most widely used wastewater treatment technologies in the world; yet, questions remain about the best strategies to improve WTL performance, particularly for nutrient removal. In this study, 1,412 facultative and upgraded WTLs were analyzed for their average effluent quality for biochemical oxygen demand (BOD), total suspended solids (TSS), ammonia (NH_3_), total nitrogen (TN), nitrate (NO_3_), and total phosphorus (TP). The geometric means of BOD, TSS, NH_3_, TN, NO_3_, and TP facility average effluent concentrations for facultative WTLs were 11.2, 19.3, 2.5, 8.8, 0.95, and 1.9 mg/L, respectively. WTLs upgraded with sand or rock filters had a lower geometric mean of average effluent BOD and TSS compared with facultative systems (Tukey–Kramer test). WTLs upgraded with moving bed-biofilm reactors, aerated gravel filters, and complete mix converted ponds showed a significant reduction in NH_3_ concentrations comparable to similar-sized activated sludge systems. A multi-variable regression analysis (*n* = 764, *R*^2^ = 0.35) found that temperature had a nonlinear effect on the average NH_3_ concentration. The nitrogen surface loading rate also had a significant, but smaller, effect. Finally, the seasonal NH_3_ variation observed in WTLs was reduced with the addition of nutrient upgrades.

## 1. INTRODUCTION

Wastewater treatment lagoons (WTLs), traditionally known as waste stabilization ponds (WSPs), are one of the oldest and most widely utilized wastewater treatment technologies in the world (EPA 2011). Most WTLs consist of large, earthen basins that rely on natural treatment mechanisms for the removal of contaminants, while others have been upgraded using various technologies (von Sperling 2007). In the United States, there are over 8,000 WTLs in operation, with approximately 4,200 discharging to surface waters (EPA 2011; Schulhof 2022). Due to their operational simplicity, low cost, and effective treatment, WTLs have been used extensively for small and rural communities. In the United States, 90% of WTLs serve communities with less than 10,000 people and treat wastewater flows less than 1 million gallons per day (MGD) (Schulhof 2022; Ruto et al. 2025).

Although the performance of WTLs has long been studied, there have been few efforts to create a comprehensive database to document and analyze their performance. The lack of an accessible database constrains our understanding of WTL operation because, although operationally simple, the complex mechanisms within WTLs (e.g., volatilization, algal-bacteria symbiosis, and anaerobic activity in the sludge layer) result in significant performance variability across facilities. While case studies provide insight into a specific system, it is difficult to conclude which factors are of most importance without comparing multiple facilities. The largest prior study, by Espinosa et al. (2017), evaluated 388 WTLs for their ability to remove biochemical oxygen demand (BOD), total suspended solids (TSS), ammonia (NH_3_), and total coliform (TC). Espinosa et al. (2017) highlighted the wide range of performance, emphasizing the effect of different pond configurations, as well as the low compliance for facilities with strict effluent limits. However, the study did not evaluate the impact of other factors and highlighted the need for further analysis of site characteristics, such as temperature and nitrogen surface loading rate, as well as other common WTL upgrades. Additional variables such as surface loading rates and temperature have been identified as having a significant effect on the performance of WTLs, yet have mostly been investigated on a case-by-case basis (Zimmo et al. 2005; Ragush et al. 2015). Consequently, further research is needed to assess the impact of upgrades, temperature, and surface loading across multiple facilities to inform future design of WTLs.

While the original design intent of WTLs was focused on wastewater stabilization (removal of BOD and TSS), many WTLs have also shown the ability to remove nutrients such as NH_3_ (Zimmo et al. 2004; Camargo Valero & Mara 2007; Bastos et al. 2018), total nitrogen (TN) (Lai & Lam 1997; Camargo Valero et al. 2010; Bastos et al. 2018), and to a lesser degree, total phosphorus (TP) (Rockne & Brezonik 2006; Powell et al. 2008). However, many of these studies have been conducted at the case study level, making it difficult to extrapolate the performance to WTL facilities with different characteristics. As nutrient regulations become more stringent in the United States, many small communities are faced with the decision to maintain the existing WTL and risk potential violation of their permit or upgrade their facility to improve nutrient removal. There are many add-on technologies for improved performance in WTLs employing mechanisms including physical filtration, such as rock filters and sand filters; physical shading, such as constructed wetlands and covers; biological treatment, such as aeration; and chemical treatment, such as ferric chloride or alum (Middlebrooks 1995; EPA 2011; Huth et al. 2021; Arslan et al. 2023). Some advanced designs utilize biofilm processes such as attached growth media filters and moving bed biofilm reactors (MBBRs) (Mattson et al. 2018; D’Aoust et al. 2022; Ruto et al. 2025). While many of the technologies have demonstrated a level of success, there have been no comprehensive performance evaluations of WTL upgrades.

The objective of this study was to create and analyze a comprehensive WTL performance database to investigate the effects of technology upgrades, temperature, and nitrogen surface loading. The work conducted builds off Espinosa et al. (2017) by utilizing a larger WTL database, testing differences between more upgrades and parameters, and evaluating the effect of site factors on performance. This study provides insight into the factors that affect the performance of WTLs and identifies the upgrade options for improving effluent quality. Practitioners may use this study as a reference when evaluating the expected performance of WTL upgrades and assessing the potential impact of climate and loading on NH_3_ performance.

## 2. METHODS

A WTL database was created containing 4,153 facilities. The effects of treatment technology, temperature, season, and nitrogen surface loading were analyzed for their impact on WTL effluent concentrations. The methods used to create the WTL inventory, categorization of technologies, and multivariate regression are described in the following sections.

### 2.1. WTL inventory

A national inventory of WTLs was developed using multiple publicly available federal and state datasets to identify and characterize facilities across the United States serving fewer than 10,000 residents or with design capacities below 1 MGD. Primary data sources included the Integrated Compliance Information System–National Pollutant Discharge Elimination System (ICIS–NPDES) and Clean Watersheds Needs Survey (CWNS) for the years 2000–2022. The sources were supplemented by the Water Pollution Search Tool, Wastewater Technology Clearinghouse, and the 2022 U.S. EPA Lagoon Inventory that integrated 18 federal and state repositories.

All datasets were merged and analyzed using R (v3.6) and Microsoft Excel 2024, with unique identifiers such as NPDES ID and facility name used to ensure consistency. The WTLs were isolated through keyword searches for terms including ‘lagoon’, ‘stabilization’, and ‘pond’. Following initial compilation, a quality control process was conducted to verify that all identified facilities represented true WTLs. First, the process description of all available NPDES permits was read to assess whether the facility utilized lagoons in its main treatment train. Systems that used lagoons for storage or equalization were not included. If the permit was not publicly accessible, satellite imagery of the facility was observed to gauge the treatment train. Given the rural context of most of the systems, it was possible to locate WTLs and basic upgrades such as aeration, rock filters, and sand filters. Out of the initial search of 4,606 potential systems, a total of 4,153 WTL facilities were identified.

The Discharge Monitoring Reports (DMR) of each facility were downloaded from the EPA ECHO Database for the date range from July 2020 to July 2024. The DMR contained all the parameters that the facility was mandated to report and included the limit values. The data did not include a description of analytical methods used, and it was assumed that either Standard Methods or other accepted techniques were used in accordance with regulatory compliance. Effluent and influent concentrations were reported monthly in various statistical bases such as ‘daily max’, ‘monthly average’, ‘30-day average’, and ‘monthly max’. The ‘monthly average’ and ’30-day average’ data were used to best represent the performance of the facility. In the cases where neither was available, ‘daily max’ concentrations were used as they represented monthly grab samples. Because most facilities are only mandated to take one sample per month, the ‘daily max’ and ‘monthly average’ are the same value, regardless of metric. Out of the various parameters, eight were selected for analysis: TSS, carbonaceous biochemical oxygen demand (cBOD), BOD, NH_3_, TN, TP, nitrate (NO_3_), and nitrite plus nitrate (NO_2_ + NO_3_). The BOD and cBOD values were combined and analyzed as one parameter due to their similar probability distributions. The NO_3_ and NO_2_ + NO_3_ values were combined due to the relatively low amount of available data. While the focus of this study was performance, the DMRs were also analyzed to determine the state of compliance and regulation for surface discharging WTLs in the United States. The results of the regulatory analyses can be found in Ruto et al. (2026).

### 2.2. Technology categorization and comparison

Based on the available information, the facilities that were confirmed to be WTLs were categorized according to their status as a facultative or upgraded treatment train. These categories included facultative (nonaerated), partially aerated, sand filters, rock filters, wetlands, covers, and chemical treatment for phosphorus removal. Additionally, three nutrient-specific technologies were located and included in the performance evaluation: (1) MBBRs, (2) aerated gravel filters (AGFs), and (3) converting the pond to a complete mixed cell followed by a fixed film nitrification reactor (CFFs). The selection of additional technologies was based on occurrence in the permit review, as the study is focused on evaluating fully operational upgrades. Whereas the other upgrades were likely implemented earlier in the WTL design, some of the nutrient-specific technologies were implemented more recently. The data for each facility were inspected to determine when the process was added and then clipped to be representative of performance following technology implementation and startup. Due to the lack of availability of historical data, an additional year (2025) was analyzed to capture data from facilities with recent nutrient-specific upgrades.

During the quality control process, 247 facilities were identified as activated sludge plants and were placed in a non-WTL category. Most of the activated sludge facilities were found to serve small communities, and many were replacements for legacy WTL systems. The activated sludge facilities were included as a separate category for comparison, as they are commonly used as a total replacement option for small communities served by WTLs.

The average effluent concentration (mg/L) of each facility was calculated using the monthly average or monthly grab sample values obtained from the DMRs. Facilities with at least 12 points of data, representing at least one year, reported in at least eight different months were included in this study to ensure the average concentration accurately summarized performance and seasonal variability. Obvious data outside reasonable expected concentrations (ranges shown in the Supplementary material, Table S1) were removed to maintain veracity. Some technologies had only a few facilities that reported data for certain parameters and were omitted if fewer than 10 facilities with data existed. The facility’s average effluent concentrations were then grouped by their respective technology categorization.

To evaluate the significance of differences between the technology groups, an analysis of variance was conducted, followed by a Tukey–Kramer test (*α* = 0.05) on the effluent concentration groups for each parameter. Due to the right-skewed distribution of the average effluent values (data shown in Figure S1), the data were natural-log transformed, resulting in a comparison of the ratio between geometric means.

### 2.3. Multiple variable regression for average NH_3_ effluent concentrations

Further analysis was conducted specifically on the facility average effluent NH_3_ concentrations to evaluate the combined significance and effect of technology type, temperature (T), and nitrogen surface loading rate (NLR). Like in the technology comparison, the NH_3_ effluent concentrations were natural-log transformed due to the right-skewed distribution. The technology groups were aggregated due to overfitting and many nonsignificant predictors, as will be discussed in the results section.

Using the coordinates from each facility, monthly average 2-meter air temperatures were downloaded using the NASA POWER database from 2020 to 2024 to match the obtained DMR data. The average annual temperature was calculated for each facility and merged with the average effluent concentrations. In addition to the regression analysis, where temperature was modeled as a quadratic, the facilities were grouped by their calculated average annual temperature values and plotted on a cumulative probability distribution for visualization. The groups that provided most clarity and distinction were <5 °C (very cold), 5–10 °C (cold), 10–15 °C (warm), and >15 °C (very warm).

The nitrogen surface loading rate (kg N/acre-day) was estimated using the population served by each facility, the surface area of the WTLs, and an assumed per-capita nitrogen contribution rate. The population of the communities served by WTL facilities with at least 12 points of data was estimated using the 2020 U.S. Census data. The surface area for each facility was estimated manually using Google Earth Pro. Due to the lack of influent NH_3_ data, it was assumed each person contributed 13.3 g of TN to the WTL influent each day (Crites & Tchobanoglous 1998; Tchobanoglous et al. 2014). The final nitrogen surface loading rate was calculated by multiplying the population and nitrogen contribution, then dividing by the surface area.

### 2.4. Seasonal variation

Further analysis was also conducted to assess the impact of temperature on the monthly time range for effluent NH_3_ data. Simple linear regressions were created between the average monthly NH_3_ concentration and temperature for each individual facility. To ensure only facilities with sufficient data were analyzed, facilities were required to have 24 data points from at least eight different months. The *p*-values for the facility temperature–NH_3_ regressions were calculated, and the Benjamini–Hochberg false discovery rate (FDR) correction method was applied (*α* = 0.05, *m* = 561) to account for Type I error in the multiple comparisons. The number of facilities with either significant or nonsignificant correlations was calculated after the FDR correction.

To evaluate the impact of seasonal variation, the average NH_3_ concentration for each month was calculated to provide an average value for each facility to ensure all systems were weighed equally. The facilities were categorized based on their annual average temperature as described in Section 2.3. Points greater than 1.5 times the interquartile range were removed to improve clarity and to eliminate outliers.

## 3. RESULTS AND DISCUSSION

The results discussed in this section include a performance comparison of facultative WTLs with upgraded WTLs and an evaluation of the impact of temperature, season, and surface loading on NH_3_ effluent concentrations.

### 3.1. Effect of technology and baseline WTL performance

**Figure 1 ∣:**
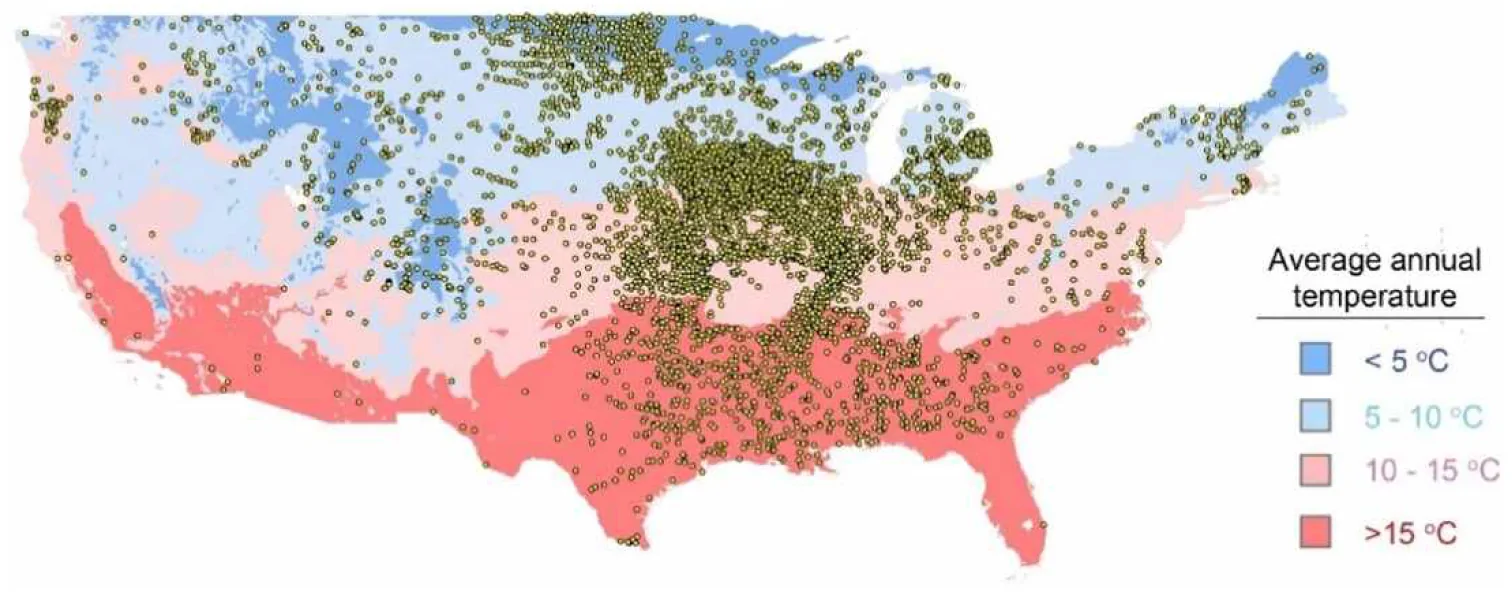
Map of WTL facilities in the United States with annual average temperature zones.

A total of 4,153 systems were identified as WTL facilities (see Figure 1), consistent with previous reports on the number of total surface-discharging WTLs in the United States (Schulhof 2022). Most of the WTLs were in the Midwest, with Iowa, Illinois, and Missouri having a combined total of 1,365 WTL systems. Most of the systems were found in the warm (1936) and very warm (792) climates (full distribution of temperature in Figure S2). There was a total of 2,720 facultative WTLs, 869 with partial aeration, 184 with rock filters, 136 with sand filters, 68 with CFF systems, 57 with AGFs, 34 with covered cells, 32 with chemical precipitation for TP removal, 28 with MBBRs, and 24 with wetlands. However, many facilities did not continuously discharge and were excluded from the performance evaluation as they did not have sufficient data available. The number of facilities per parameter that met the criteria outlined in the methods is shown in Table 1. There were a total of 1,412 unique facilities that were evaluated. Technologies that had geometric means of the average effluent concentrations significantly different (Tukey–Kramer test, *α* = 0.05) from aerated and facultative systems are also shown in Table 1. The full list of significant Tukey pairs can be found in Tables S2-S7. All technologies had a relatively high geometric standard deviation for each parameter, indicating that there are likely other factors affecting performance besides technology type.

It should be noted that the performance evaluation only considered surface discharging WTLs, which are regulated by the NPDES system. There are approximately 4,000 additional WTL systems that land-apply their effluent through methods such as percolation basins, evaporation basins, and spray irrigation (EPA 2011). Future work could involve evaluating WTLs characterized as land application type discharge and determining if their performance is consistent with the WTLs analyzed in this study.

#### 3.1.1. BOD

**Figure 2 ∣:**
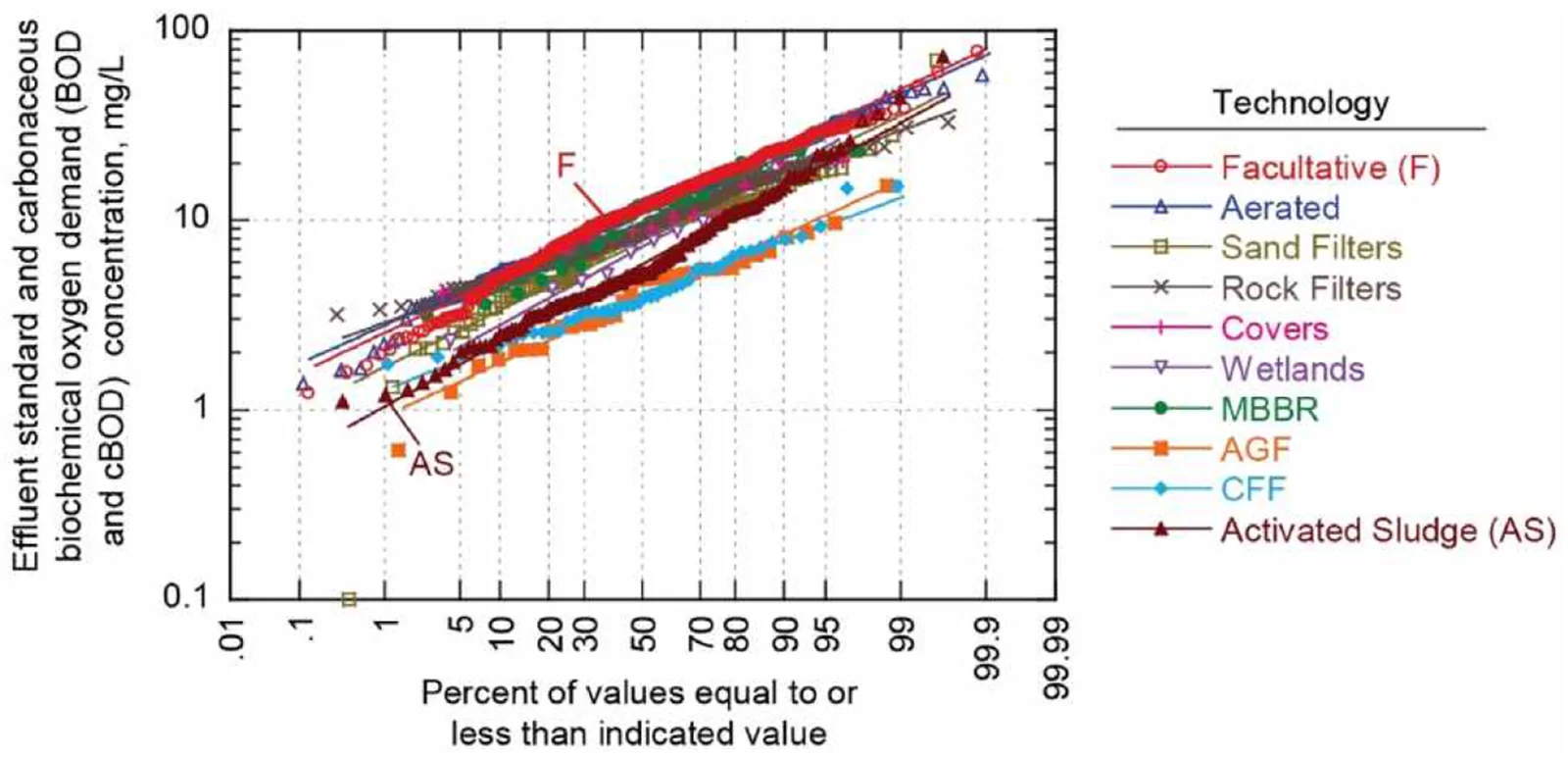
Probability distribution of the BOD effluent for WTLs categorized by technology.

The BOD and cBOD values were combined due to their similar probability distributions. The BOD values were found to be approximately 3 mg/L higher than cBOD across all technologies. Facultative WTLs had the highest average effluent BOD concentration (Table 1). Ponds equipped with various aeration systems did not improve effluent quality (Figure 2) despite literature demonstrating aeration to be effective at reducing BOD concentrations in WTLs (Vagheei 2021). It should be noted that the level of aeration was not known for each facility and that the aerated WTLs analyzed in this study may behave as partially mixed facultative systems and not fully mixed aerated systems. By comparison, activated sludge systems showed a significant reduction in BOD concentration, with the geometric mean of the average concentrations being 48% lower than that of facultative systems.

Covers and wetlands had slightly lower geometric means for BOD concentration compared with facultative systems but were not statistically significant due to the low sample sizes. Rock filters and sand filters reduced the geometric mean of average BOD concentrations by 17 and 31%, respectively, compared with facultative systems. Both rock filters and sand filters had geometric means significantly different than facultative or aerated systems. Most of the rock filters were located in Illinois, where a previous evaluation had found similar BOD performance (Middlebrooks 1988). The majority of sand filters were intermittent type and have also previously been demonstrated to improve BOD effluent (Harris et al. 1977; Middlebrooks 1995).

The AGF, CFF, and MBBR implementations reduced the geometric mean of average BOD concentrations by 65, 63, and 13%, respectively, when compared with facultative systems. Unlike AGF and CFF, the geometric mean of BOD effluent concentrations for MBBRs was not significantly different than facultative or aerated systems, potentially due to their intended design focusing on NH_3_ removal. The AGF and CFF upgrades had the lowest BOD concentrations, highlighting their ability to remove both soluble and particulate forms of BOD from WTL effluent.

#### 3.1.2. TSS

**Figure 3 ∣:**
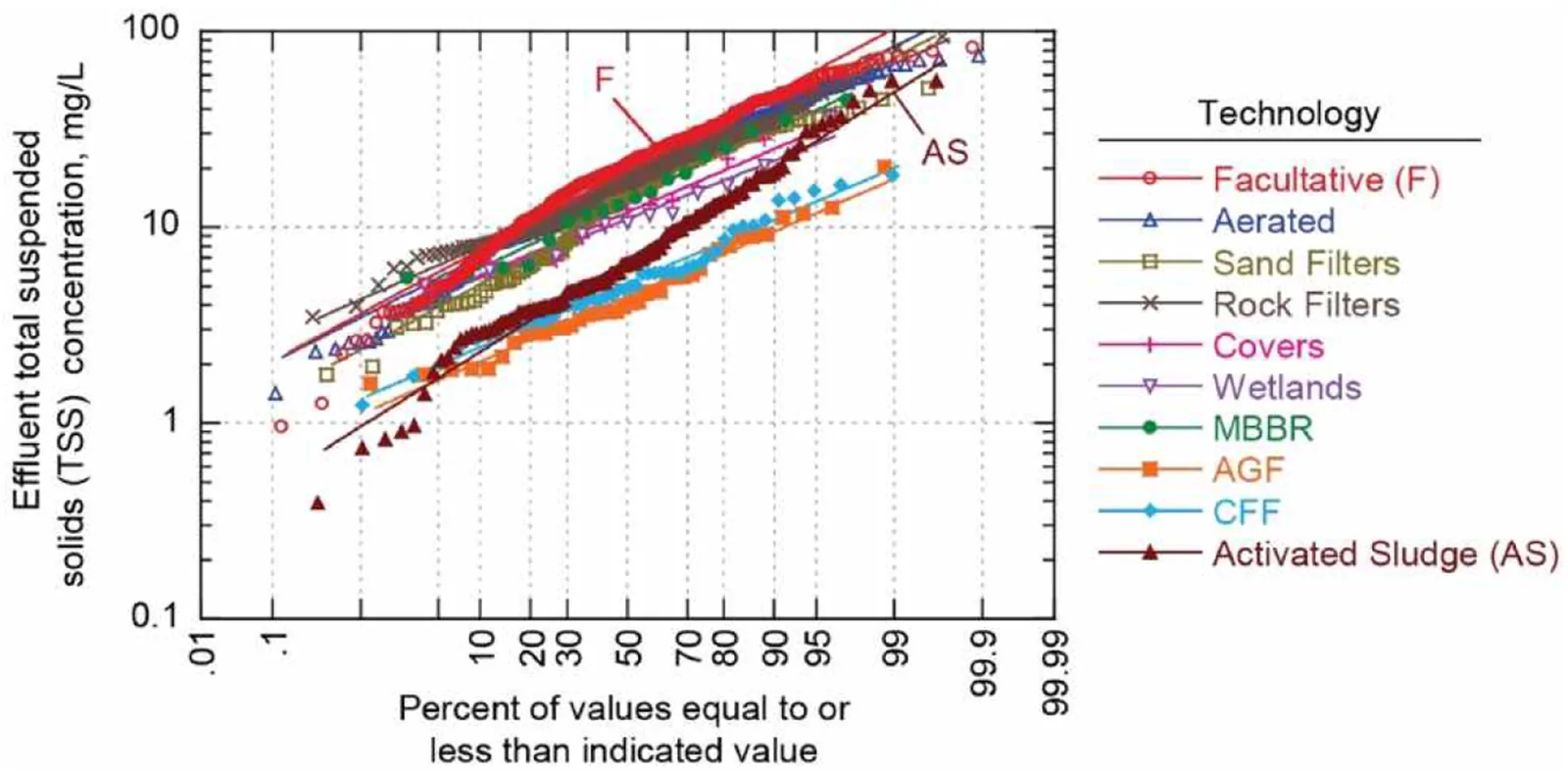
Probability distribution of the TSS effluent for WTLs categorized by technology.

The geometric mean of average TSS effluent concentrations for facultative systems was the highest among all technology options (Table 1). Facultative WTLs often experience algal blooms that result in elevated BOD and TSS concentrations (EPA 2011). The geometric mean of average TSS concentrations for aerated WTLs was slightly lower than that of facultative systems but not significantly different (Figure 3). Covered WTLs and wetlands also reduced the geometric mean of TSS by 38 and 43%, respectively, but were not statistically different than facultative systems due to their small sample size. As previously mentioned, both covers and wetlands can prevent algae growth, lowering overall TSS concentrations, but the limited data in this study prevent the finding of a statistically significant difference compared with baseline systems.

Sand filters improved the geometric mean of average effluent TSS concentrations by 32% when compared with facultative systems. Rock filters were less effective but still improved the TSS concentration by 11%. Both rock filters and sand filters had significant differences in geometric mean when compared with aerated and facultative systems. The differences agree with the literature, which reports that both sand filters and rock filters are able to remove algae from WTL effluent and are a relatively low-cost upgrade option for facilities struggling with algal blooms (Harris et al. 1977; Middlebrooks 1995).

The AGF, CFF, activated sludge, and MBBR systems reduced the geometric mean of average effluent TSS concentrations by 77, 73, 65, and 28%, respectively, compared with facultative systems. Each of the listed technologies had a significant difference except for MBBRs. The AGF and CFF had the lowest TSS concentrations on average. Activated sludge TSS effluent concentrations were far lower than those of facultative systems, as expected, as the addition of dedicated clarifiers usually results in more effective solids treatment. Overall, medium-level upgrades such as sand filters and rock filters reduced TSS significantly compared with facultative ponds but can be further improved by the advanced upgrade systems.

#### 3.1.3. NH_3_

The relatively low NH_3_ concentrations (Table 1) of facultative systems are consistent with case studies that have reported up to 90% removal (Bastos et al. 2018). Despite originally being designed for BOD and TSS removal, facultative systems, on average, seem capable of achieving low NH_3_ concentrations (geometric mean of average NH_3_ concentrations = 2.4 mg/L) either through volatilization or algal assimilation (Rockne & Brezonik 2006; Camargo Valero & Mara 2010). However, facultative systems also had significant NH_3_ variability (geometric standard deviation = 2.9 mg/L), consistent with previous performance evaluations (Espinosa et al. 2017). The effect of other factors on NH_3_ effluent concentrations will be discussed in a later section, but it should be noted that because most of the facilities are in warmer climates, the geometric mean is more representative of warm temperature conditions. Aerated facilities had a higher geometric mean of average NH_3_ concentrations and standard deviation than facultative WTLs. While theoretically sufficient aeration could be supplied to inhibit algae growth and nitrify incoming NH_3_, most systems do not seem to achieve full nitrification and operate more as partial-mix systems. However, the elevated NH_3_ concentrations in aerated facilities compared with facultative systems are confounded by the absence of data regarding site-specific aeration usage.

Wetlands had limited NH_3_ data with a geometric average concentration higher than that of facultative systems, while covers had insufficient data for analysis. Covers are not expected to improve the removal of soluble nutrient species, such as NH_3_, and are mostly for reducing algae growth. However, wetlands have been demonstrated to reduce WTL effluent NH_3_, even in cold temperatures (Huth et al. 2021; Arslan et al. 2023). The differences in geometric means for wetlands and facultative WTLs were not significant, and additional data are needed to accurately characterize the capabilities of well-defined constructed wetlands.

Sand filters and rock filters did not significantly reduce the geometric mean of average effluent NH_3_ concentrations when compared with facultative systems. Previous studies on WTL systems upgraded with rock filters in Illinois found that while filters can remove significant TSS and BOD, nonaerated implementations did not effectively remove NH_3_ (Middlebrooks 1988). Other studies have demonstrated that rock filters can improve NH_3_ removal in WTL effluent if they are equipped with proper aeration (Archer & O’Brien 2005; Johnson & Mara 2005). Therefore, the absence of a difference between rock-filter upgraded and facultative WTLs is likely due to most rock filters being passive, nonaerated systems.

**Figure 4 ∣:**
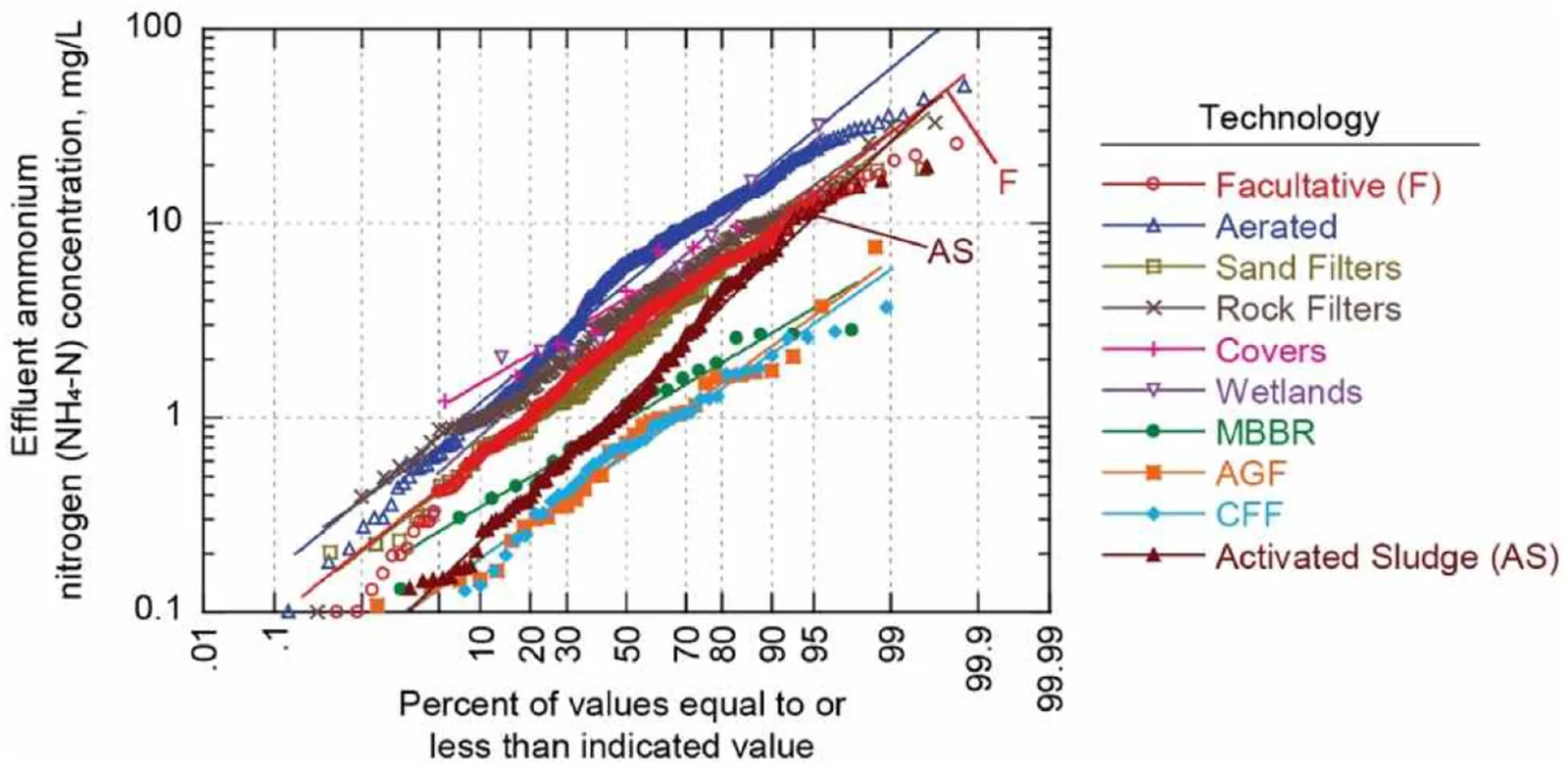
Probability distribution of the NH_3_ effluent for WTLs categorized by technology.

The geometric mean of average effluent NH_3_ concentrations for CFF, AGF, and MBBR was 84, 73, and 59% lower than that of facultative systems (all significant), respectively. The three nutrient technologies also had smaller standard deviations, making them more reliable across their implementations. Additionally, all three technologies had a geometric mean below 1 mg/L (Figure 4), which has been found to be a common limit for WTL facilities (Ruto et al. 2026). The geometric means were also similar to those of activated sludge systems despite maintaining the existing lagoon infrastructure in the treatment train, although the lack of activated sludge information regarding sludge retention time, configuration, dissolved oxygen, and loading limits the comparison.

#### 3.1.4. TP

**Figure 5 ∣:**
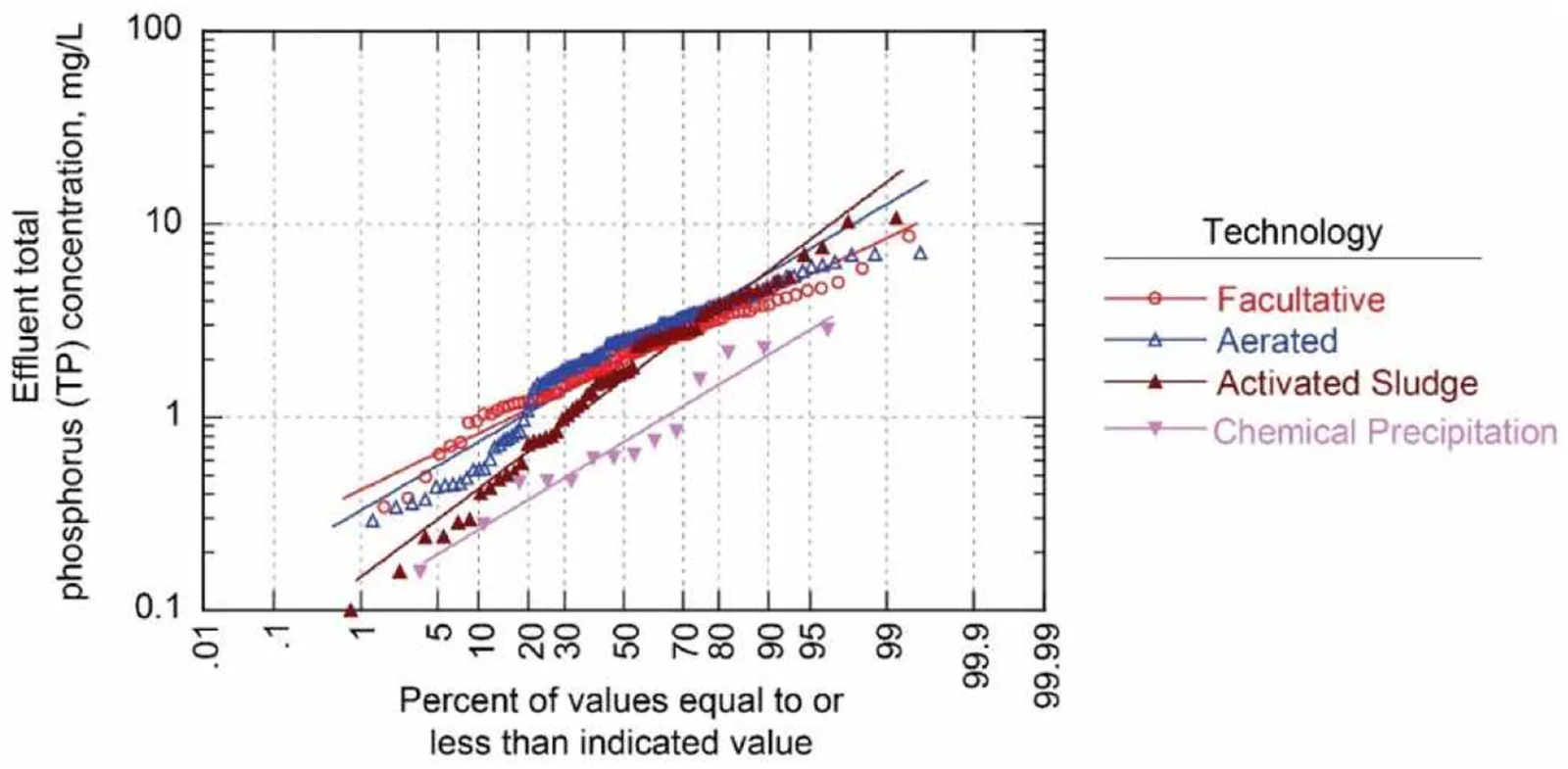
Probability distribution of the TP effluent for WTLs categorized by technology.

The most effective treatment for TP among all the technologies examined was chemical dosing (Figure 5). Among the 32 facilities identified, 17 used ferric chloride to reduce TP in their effluent, although only 14 WTLs were analyzed. Chemical precipitation of phosphorus is well known and utilized in wastewater treatment, with other chemical options including aluminum sulfate. Chemical precipitation was the only technology evaluated that had a geometric mean of average TP effluent concentrations below 1 mg/L, which has been identified as the most common limit for TP permitted WTL facilities (Ruto et al. 2026). The geometric mean of the chemical precipitation group was significantly different than the baseline groups, although the sample size was much smaller. Despite the low count, the result is consistent with the well-established use of chemicals for TP removal. Facultative and aerated systems had higher effluent TP concentrations, consistent with other literature reporting only 30–50% removal (Rockne & Brezonik 2006). Activated sludge systems had slightly better performance and are capable of being operated to target TP removal through enhanced biological phosphorus removal.

#### 3.1.5. NO_3_

**Figure 6 ∣:**
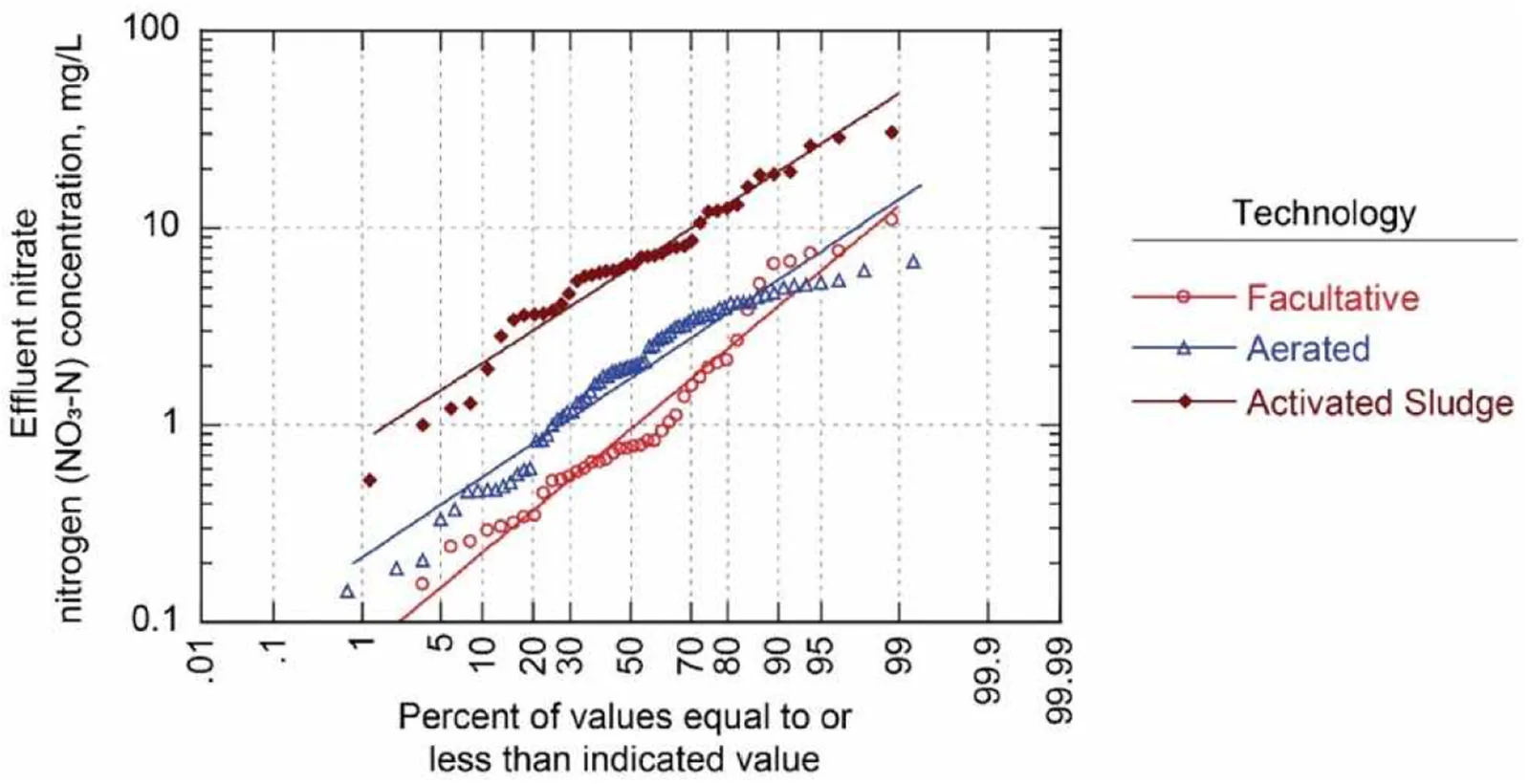
Probability distribution of the NO_3_ effluent for WTLs categorized by technology.

Facultative systems had low nitrate concentrations (Figure 6), although less data were available compared with other parameters, consistent with other studies that have found only low levels of nitrate formation in facultative systems (Camargo Valero et al. 2010; Bastos et al. 2018). However, it should be noted that the absence of NO_3_ concentrations does not necessarily mean nitrification does not occur, as NO_3_ could be removed by denitrification or algal uptake. Aerated ponds had a significantly higher geometric mean of average nitrate concentration compared with facultative systems, as some facilities are likely to operate at the right conditions to grow nitrifying biomass. However, as demonstrated by the NH_3_ data, the geometric mean of average effluent NH_3_ concentrations in aerated WTLs was higher than in facultative systems. Activated sludge systems had the highest NO_3_ concentrations as expected, implying that most systems are capable of reliable nitrification. There were insufficient data available to evaluate the performance of other upgrades.

#### 3.1.6. TN

**Figure 7 ∣:**
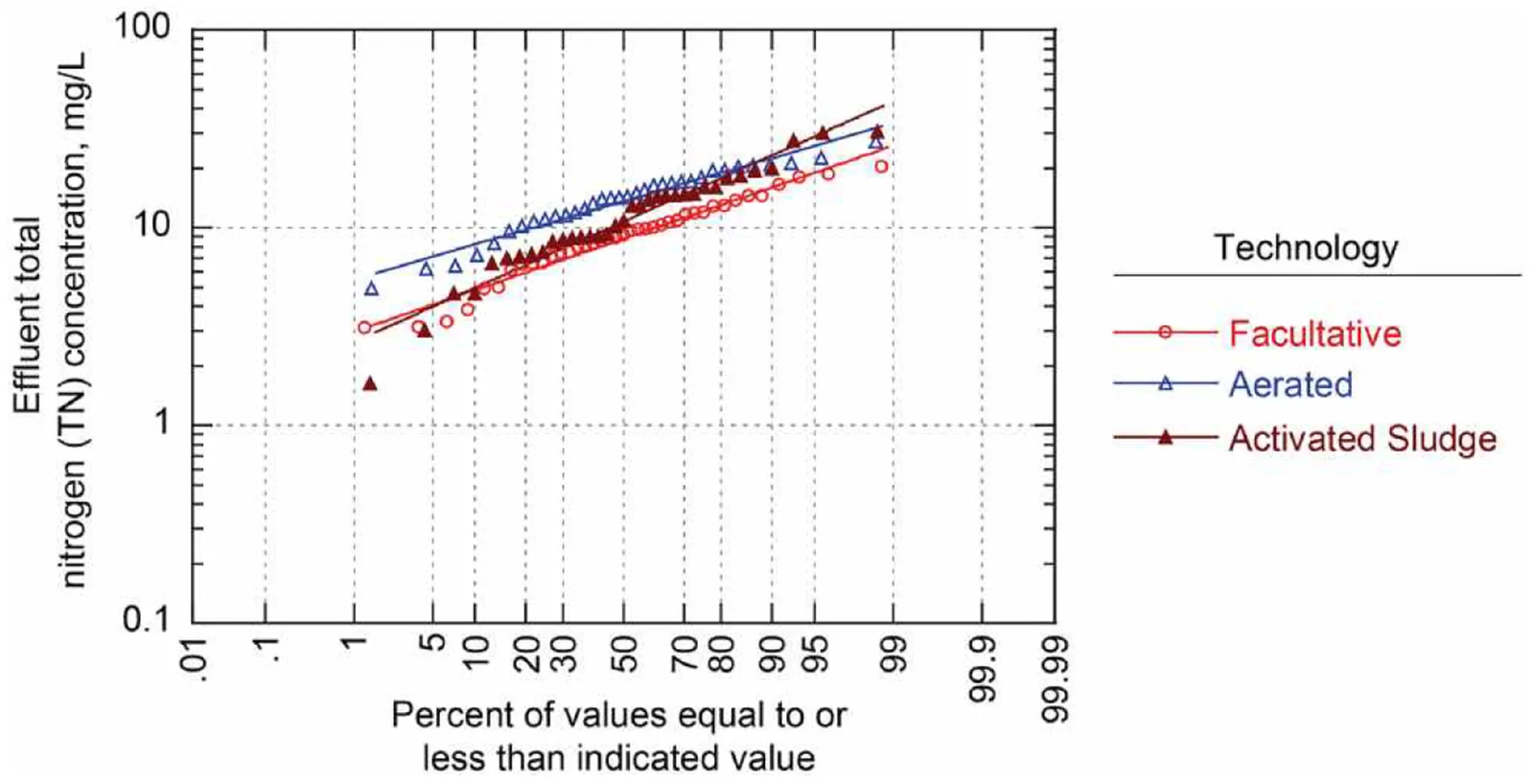
Probability distribution of the TN effluent for WTLs categorized by technology.

Limited TN data were only available for facultative, aerated, and activated sludge systems, which had high geometric standard deviations. Facultative systems had the lowest geometric mean of average concentrations yet had the highest geometric standard deviation (Figure 7). Previous studies have demonstrated significant TN removal through NH_3_ volatilization and settling of organic nitrogen, although the dominant mechanism is still debated (Rockne & Brezonik 2006; Bastos et al. 2018). Aerated systems had a higher geometric mean of average concentrations and considerable variation as well. The main conclusion regarding the TN performance is that more data are needed to provide a more concrete evaluation. The small dataset (only 79 WTLs from two technology groups analyzed) is insufficient to draw conclusions about the effectiveness of WTLs or upgrade technologies.

### 3.2. Combined effects of technology, temperature, and nitrogen surface loading rate on ammonia concentrations

While the previous section shows the differences between technology groups for various parameters, there are several other factors that can affect the effluent concentrations of wastewater lagoons. Given increasing nutrient limits, it is important to determine the effect size and significance of other factors on nutrient removal. NH_3_ was selected for further investigation due to the emphasis on this study on nutrients and sufficient effluent concentration data being available. A multivariable regression was performed on the average NH_3_ effluent concentrations using average annual temperature, nitrogen surface loading rate, and technology as predictors.

#### 3.2.1. Multi-variable regression construction

The final multivariable regression utilized can be seen in Equation (1) and the summary of coefficients in Table 2. As was shown in Section 3.1, the NH_3_ effluent concentration data were not normally distributed and were natural log transformed. The *Q*–*Q* plots and standardized residuals confirm the linearity of the model after transformation and can be found in Figure S3. The residuals and fitted values were also plotted and demonstrated no evidence of heteroscedasticity (Figure S3). Temperature was modeled as a quadratic function due to its effect relying on the nonlinear (Arrhenius equation) relationship between temperature and microbial growth rate (Huang et al. 2011). The quadratic regression was superior to previously tested linear versions, as the quadratic term was significant and improved the overall explanatory power of the model. Multicollinearity was tested using the general variable inflation factor (GVIF). Due to the addition of the quadratic term and the lack of meaning when the intercept for surface loading was zero, temperature and surface loading were centered around their medians, which reduced multicollinearity to acceptable levels (GVIF <2, Table S8). The effect of interactions between each variable (temperature × surface loading, surface loading × technology, and technology × temperature) was tested in larger models but was found not to significantly affect NH_3_:

(1) $$\text{ln}\;({\text{NH}}_{3})={\hat{\beta }}_{0}(\text{technology})+{\hat{\beta }}_{1}T+{\hat{\beta }}_{2}T^{2}+{\hat{\beta }}_{3}\text{NLR}$$

Initial regression attempts were conducted using all the technologies presented in Section 3.1 as categorical variables but resulted in over-fitting and many nonsignificant factors. To reduce the complexity of the model while maintaining realistic expectations of technology performance, the technologies were combined into larger groups based on their expected ability to remove NH_3_. The ‘basic’ group (*n* = 216), which was used as the reference for the regression, consisted of facultative WTLs, covers, and chemical phosphorus removal, as the upgrades were not expected to contribute to NH_3_ performance. Aerated lagoons (*n* = 241) were maintained as their own group due to their potential to nitrify but again were limited due to the unknown level of aeration. The ‘mid-level’ group (*n* = 222) consisted of sand filters, rock filters, and wetlands, as they are technologies known to improve NH_3_ effluent, but only under appropriate conditions. Finally, the ‘advanced’ technology group (*n* = 76) included MBBRs, AGFs, and CFFs as they were expected and designed to improve NH_3_ effluent.

#### 3.2.2. Explanatory power and effect of factors

**Figure 8 ∣:**
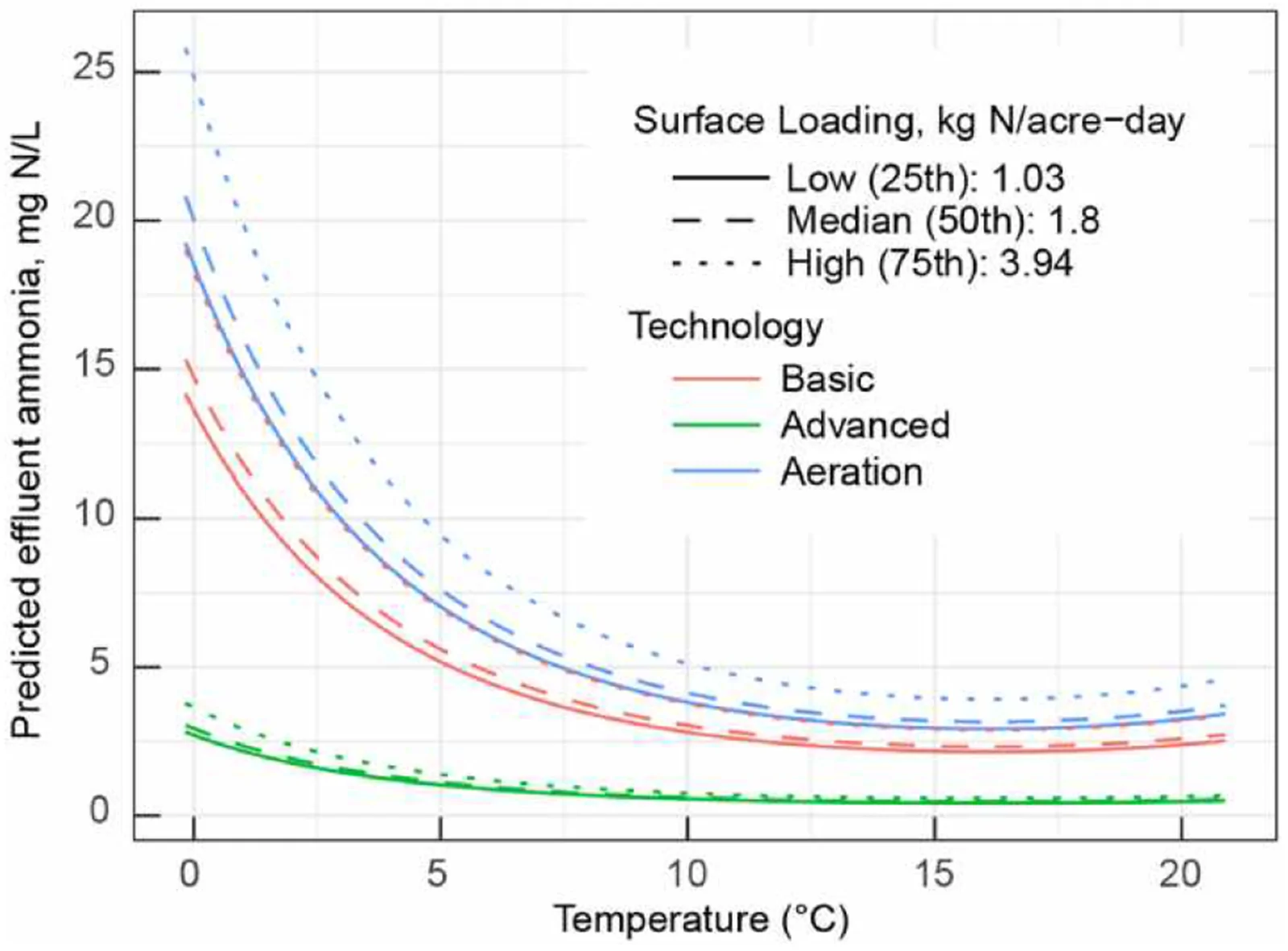
Predicted average NH_3_ concentration based on regression (see Equation (1)) estimated by technology type, temperature, and nitrogen surface loading rate (corresponding to the 25th, 50th, and 75th percentile).

The adjusted *R*^2^ = 0.35 suggested overall weak explanatory power and that additional variables were missing. Other factors that could influence the effluent concentration of NH_3_ from lagoons include solar radiation, depth, aeration input (for aerated systems), algae species, wind, and more. However, even though the model could be improved with additional variables, the regression still provides insight into the relationship of predictors with NH_3_ effluent concentrations. In Figure 8, the predicted NH_3_ effluent concentration (back-transformed to linear scale for improved interpretability) of lagoon facilities is plotted as a function of the predictors used in the regression.

The ‘basic’ technology group, which represented mostly facultative systems, was used as a reference for the model. The ‘advanced’ group had a significant effect (*p* < 0.0001) and a coefficient value of −1.61, equating to reducing the average effluent concentration by 80% when temperature and surface loading are at their median values. The ‘mid-level’ group had no statistically significant effect on the average effluent concentration of a facility. The ‘aerated’ group significantly affected NH_3_ but increased the concentration, similar to what was seen in Section 3.1. One interpretation could be that aeration units are often added to increase BOD removal capacity and that the influent nitrogen concentrations for aerated facilities were higher than typical literature values used in the regression. Overall, the effect of technologies was the same as seen in Section 3.1, where advanced technologies significantly improve effluent quality, aerated are statistically higher, and mid-level upgrades do not change performance.

As described in Section 3.2.3, the quadratic modeling of temperature was statistically significant and coincides with the nonlinear effect of temperature on microbial growth, although it does not exactly mimic the Arrhenius equation (Huang et al. 2011). Both the linear and quadratic terms were significant in predicting the effluent ammonia concentration with coefficients of −0.0527 and 0.0071, respectively. The predicted relationship between temperature and effluent NH_3_ can be seen in Figure 8. Colder temperatures result in lower effluent NH_3_ concentrations with diminishing effects until 16 °C, where the relationship switches. Given that microbial growth is driving NH_3_ removal, especially algae in facultative systems, it makes sense that the relationship is very similar to the inverse of temperature-algae growth rate curves, where the growth rate can decrease after 15 or 20 °C (Nalley et al. 2018).

**Figure 9 ∣:**
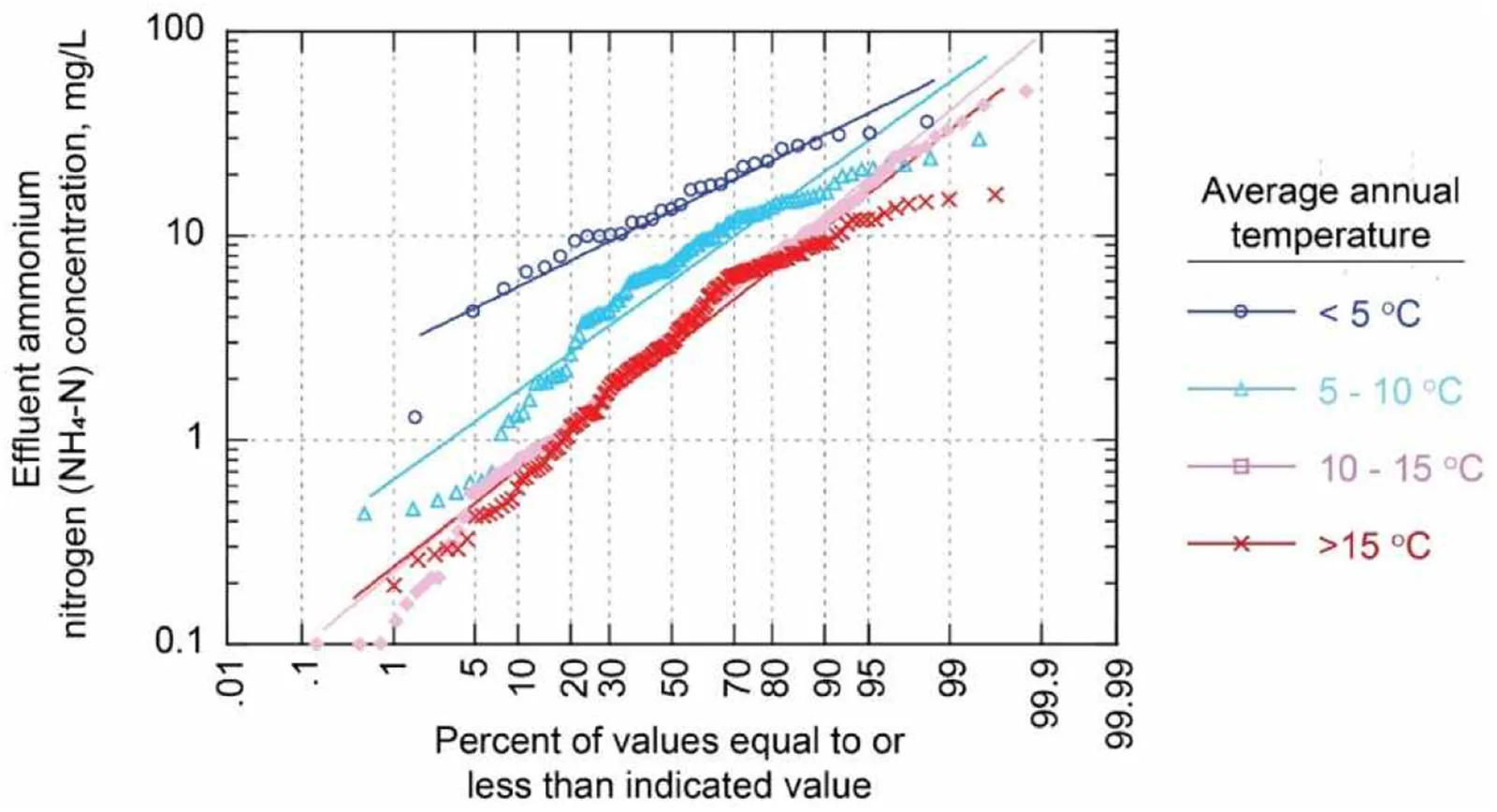
Cumulative probability distribution of average NH_3_ concentrations for aerated and facultative systems categorized by temperature groups.

The effect of temperature can also be visualized through cumulative probability distributions. In Figure 9, the average effluent NH_3_ concentrations for facultative and partially aerated lagoons were plotted on a cumulative probability distribution categorized by their annual average temperature. The four groups selected for visualization purposes were 0–5 °C (very cold), 5–10 °C (cold), 10–15 °C (warm), and >15 °C (very warm). Clearly, the facilities in the very cold and cold groups had higher effluent NH_3_ concentrations, with geometric means of the average concentrations being 13.2 and 8 mg/L, respectively. The high NH_3_ concentrations demonstrate that facilities in especially cold climates are at higher risk for violating even lenient NH_3_ limits (i.e., 10 mg/L). The warm and very warm groups had lower geometric means of 3.1 and 2.8 mg/L, respectively. The results in Figure 9 demonstrate an alternative visual to the diminishing benefit of temperature seen in the quadratic relationship found in the regression (Figure 8).

The final variable tested for its effect on the average effluent NH_3_ concentration of lagoons was the nitrogen surface loading rate. The nitrogen surface loading rate was an attempt to gauge the sizing of a lagoon compared with its mass loading in the absence of retention time or influent characterization data. It should be noted that the variable has the most uncertainty among the factors, as the nitrogen loading rate was calculated based on the served population and an assumed production of 13.3 g of TN per person. In the regression, the main effect of surface loading was significant, and there were no significant interactions with temperature and surface loading. While significant, the overall effect size is relatively small, as can be observed in Figure 8. In theory, higher nitrogen surface loading rates, where the lagoon has a lower retention time compared with the nitrogen mass loading, should result in worse treatment. However, the effect seems relatively minor compared with the temperature and upgrade type.

Overall, the results of the multivariable regression demonstrate that technology type, temperature, and nitrogen surface loading rate have a significant effect on the average NH_3_ from a WTL facility. The low adjusted *R*^2^ of 0.35 suggests that many other variables are missing and causes uncertainty about the effect size of the analyzed factors. Still, the regression demonstrates that WTLs with advanced upgrades can achieve relatively low NH_3_ concentrations even at lower temperatures and higher loading rates. The inverse relationship of temperature on effluent concentration is significant and consistent with the literature, while the effect of surface loading rates is significant but smaller.

### 3.3. Seasonal variation of NH_3_ effluent concentrations

The previous exercise evaluated the effect and relationship of factors on the overall annual average NH_3_ effluent concentration. However, WTLs are also known to have very seasonal performance, mostly due to temperature change (Smyth et al. 2018). The following sections discuss the prevalence of seasonal NH_3_ performance of lagoons and the benefit of adding advanced upgrade technologies.

#### 3.3.1. Individual facility regressions for facultative and aerated systems

Facultative and aerated facilities that had sufficient monthly data for parameters were selected to create regressions with their own respective monthly temperatures. The *p*-values for the facility level regressions between temperature and NH_3_ were calculated, and the FDR correction method (false discovery rate = 5%) was applied to determine the number of significant relationships.

**Figure 10 ∣:**
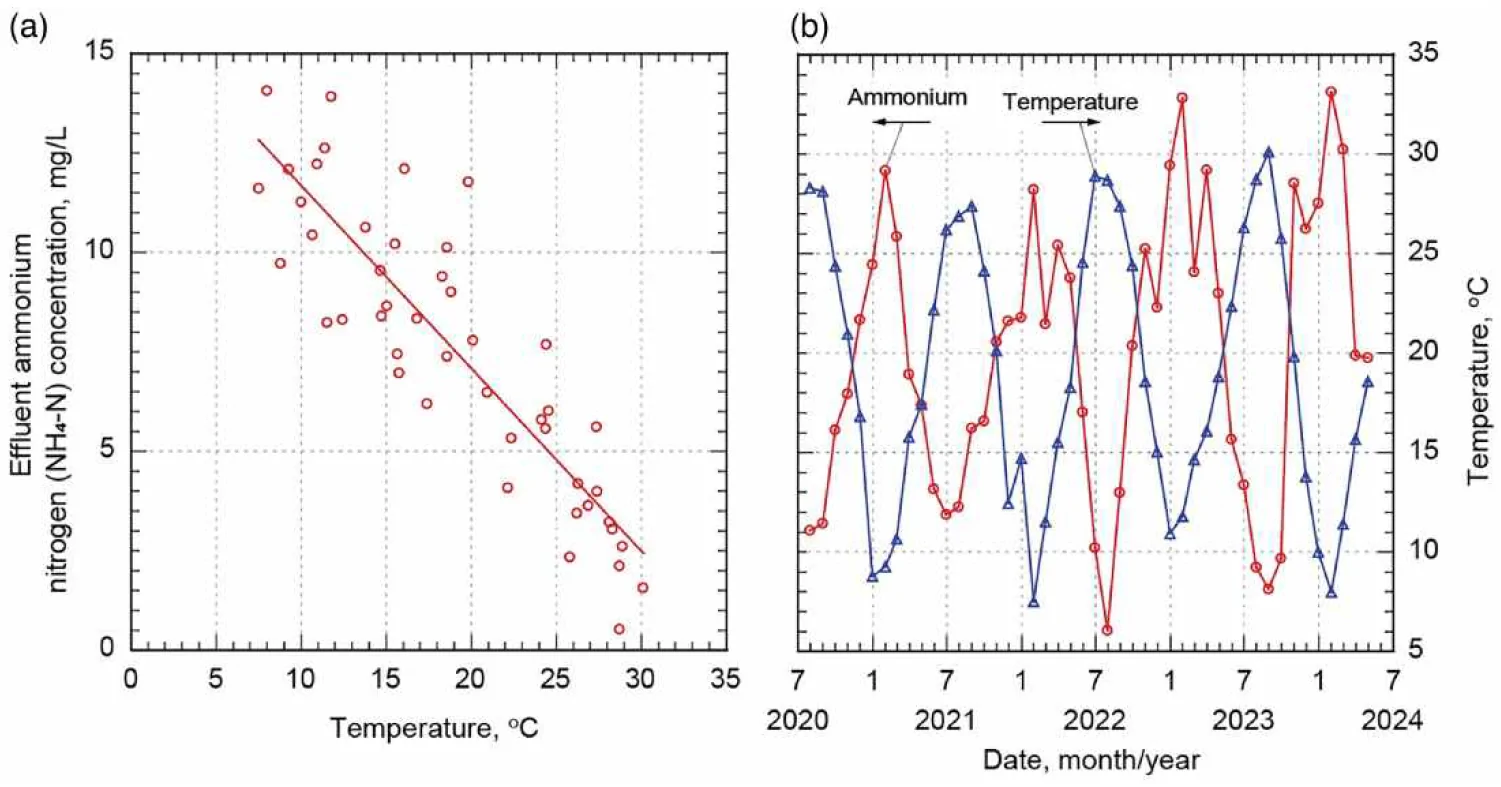
Sample data for a facultative WTL in Alabama: (a) linear regression for monthly NH_3_ and temperature and (b) a time-series plot of NH_3_ concentration and temperature for the same facility.

It was found that 284 out of the 561 analyzed facilities had a significant correlation between monthly temperature and monthly NH_3_ effluent concentration after the FDR correction was applied. Most facilities had an inverse relationship with temperature, implying that warmer months had lower NH_3_ concentrations and highlighting the seasonality of NH_3_ removal. An example of one of the facilities (located in Alabama) that had a significant correlation with temperature is shown in Figure 10(a) and 10(b), demonstrating how warmer months had lower NH_3_ concentrations compared with cold months.

**Figure 11 ∣:**
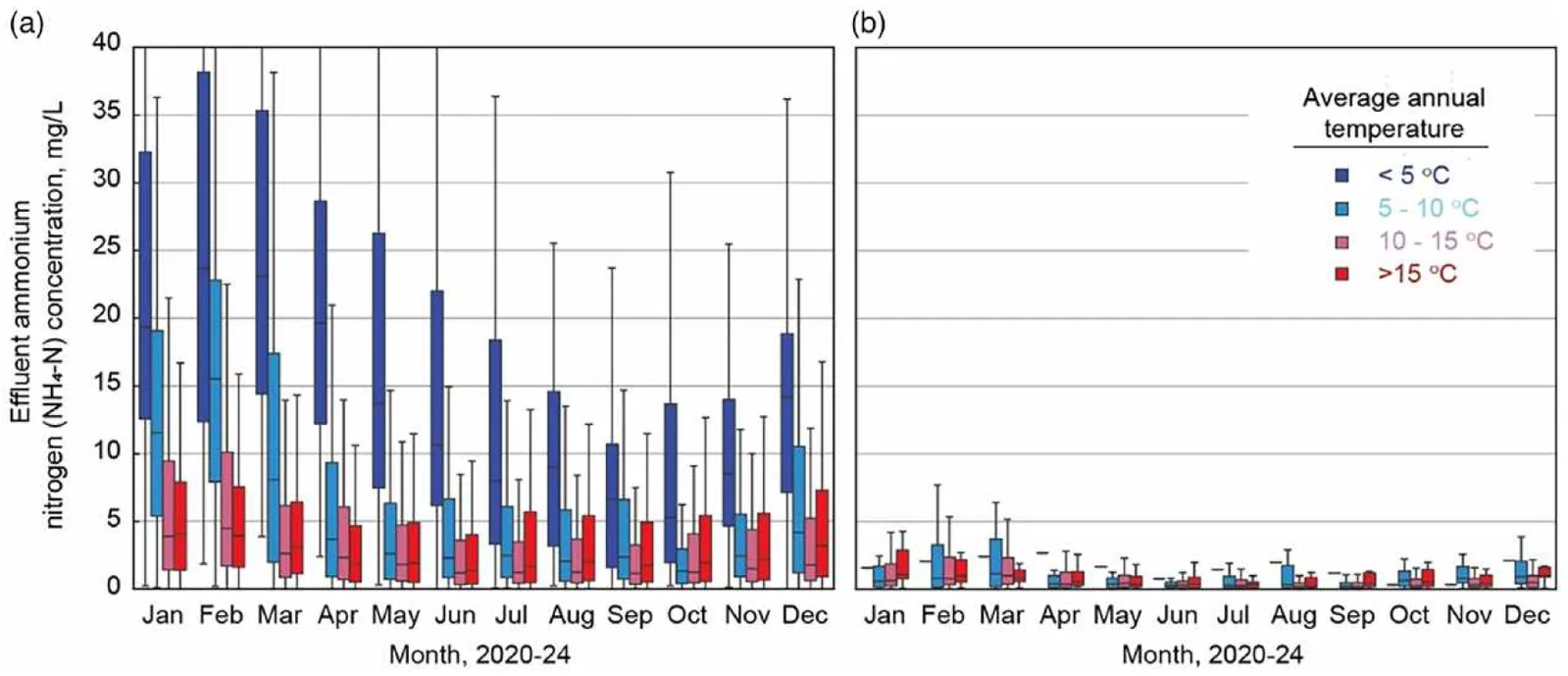
Distribution of average monthly NH_3_ concentrations grouped by annual average temperature for (a) aerated and facultative WTLs and (b) WTLs upgraded with either moving bed-biofilm reactors, complete mix ponds with fixed film nitrification, or aerated gravel filters.

To further visualize the seasonality, the average ammonia concentration for aerated and facultative systems was calculated for each month in the year (January–December). Additionally, the facilities were kept in the previously mentioned temperature groups to visualize the effect of temperature and season. The results are shown in Figure 11(a). The process was repeated for facilities with upgraded nutrient technologies [MBBR, AGF, and CFF, see Figure 10(b)].

The seasonality of NH_3_ concentrations is shown in Figure 10(a), where summer months had lower ammonia concentrations and winter months had higher concentrations. Very cold (<5 °C) facilities had a dramatic difference in average ammonia concentrations. In January, the average concentration was 22 mg/L, which rose slowly in February and March. The average concentration then decreased between April and September, with the lowest month having 9.5 mg/L of NH_3_. The concentration then decreased during the subsequent winter months (October–December).

The cold (5–10 °C) temperature group also followed the same pattern as the very cold group, with the average ammonia concentration increasing between January and March, before decreasing between April and October. While the ammonia concentrations were elevated in winter months, the summer months had similar effluent quality to the warm (10–15 °C) and very warm (>15 °C) temperature groups. It can, therefore, be expected that facultative and partially aerated WTLs will see a decrease in effluent NH_3_ concentrations during the summer, except only in the most extreme cold locations (<5 ° C). In the warmer temperatures, both the warm (10–15 °C) and very warm (>15 °C) categories had low ammonia concentrations in the effluent during the summer months, with slightly elevated levels in the winter. Very warm (>15 °C) facilities had a minimal drop in performance and had low ammonia concentrations year-round.

The nutrient upgrade WTL facilities (Figure 11(b)) had almost no seasonal variation in any of the temperature groups, although no data were available in the very cold temperature range. Despite a smaller sample size, the results showed that the three upgrades achieve lower NH_3_ effluent concentrations and are unaffected by winter months, where facultative and aerated systems saw a drop in performance. The upgrades were designed with the intention of cold NH_3_ reduction. The MBBRs were mostly designed by a manufacturer who incorporated a heating element in the reactors to achieve nitrification even at freezing temperatures. The AGFs were underground and insulated, allowing for cold-temperature NH_3_ removal as well. Finally, the CFF systems used insulated covers to elevate the temperature of the water column. All three technologies demonstrated excellent NH_3_ removal in both summer and winter conditions. The results are consistent with the regression conducted in Section 3.2, which demonstrated the ability of advanced upgraded WTLs to achieve relatively low NH_3_ concentrations (<5 mg/L) even in cold climates.

### 3.4. Limitations and future work

While comprehensive, the performance evaluation conducted in this study had several limitations that could be addressed in future work. While over 4,000 facilities were identified, only 1,412 facilities had sufficient effluent discharge data to be evaluated. Future work could include expanding the performance range or investigating the performance of intermittent discharging WTLs. In addition to the approximate remaining 2,700 WTLs that discharge to surface water, another 4,000 WTL systems that apply their effluent to land have not been evaluated. Whether the performance of the land-application WTL systems is similar to the surface-discharging WTLs remains a question, but it is expected that the mechanisms would be similar. It is notable that many of the WTLs that discharge to land are located in warm and arid climates characterized by high rates of evaporation.

The lack of operational information was also a major limitation of this study, as only general information about a facility’s treatment train and climate was used. The operation of WTLs and their upgrades undoubtedly affects performance but was not captured in this evaluation. In future work, the inclusion of site-specific lagoon details such as hydraulic retention time, pond depth, dissolved oxygen concentrations, mixing intensity, and sludge age could improve the evaluation and identify additional key factors affecting performance. The addition of the variables would likely benefit the multiple variable regression and improve its explanatory power (current *R*^2^ = 0.35) and lower uncertainty regarding the effect sizes. The use of air temperature in place of available water temperature also introduced uncertainty in the analysis.

Finally, limited data were available for influent wastewater concentrations, and assumed concentrations were used in the nitrogen surface loading analysis, which did not, for example, capture the effects of potential infiltration and inflow, or contributions from commercial and industrial sources. The lack of influent characterization constrains conclusions regarding overall removal of constituents, and the analysis primarily relied on effluent concentrations as a measurement of performance. Future work should focus on improving data acquisition or estimation techniques for influent concentrations and expand the analysis to include other nutrients, such as TN and TP.

## 4. CONCLUSIONS

In this study, a comprehensive performance evaluation of WTL facilities was conducted by developing a database of NPDES compliance data. The effect of technology, temperature, and nitrogen surface loading rate was analyzed. Facultative WTLs had relatively low effluent concentrations for parameters such as BOD, TSS, NH_3_, TN, NO_3_, and TP with geometric means of the facility averages of 11.2, 19.3, 2.4, 8.8, 0.95, and 1.9 mg/L, respectively. However, variability was high for all parameters. Basic WTL upgrades, including sand filters and rock filters, significantly lowered BOD and TSS effluent concentrations, but did not significantly lower nutrient concentrations. The nutrient-specific WTL upgrades, such as MBBRs, AGFs, and CFFs, reduced NH_3_ effluent concentrations, comparable to similarly sized activated sludge systems, highlighting these technologies as potentially effective alternative solutions for improving NH_3_ performance. The multiple variable regression revealed technology type, temperature, and nitrogen surface loading rate to significantly affect the average NH_3_ of a WTL facility, although it is likely many variables were missing from the analysis (*R*^2^ = 0.35). Temperature had an inverse quadratic effect, while the nitrogen surface loading rate effect was relatively small. Considerable seasonal variation in NH_3_ monthly data was observed, likely caused by a change in temperature. In both regression and evaluation of seasonal variation, advanced upgrades (MBBRs, AGFs, and CFFs) demonstrated the ability to achieve low NH_3_ concentrations in cold climates.

## Supplementary Material

Supplementary Information

## Figures and Tables

**Table 1 ∣:** Performance of baseline WTLs compared with upgraded WTLs

Technology (# of analyzed facilities)	Geometric mean ± geometric standard deviation, mg/L (number of facilities analyzed)					
	BOD	TSS	NH_3_	TP	NO_3_	TN
Facultative (413)	11.2 ± 1.9 (384)	19.3 ± 2.1 (385)	2.4 ± 2.9 (274)	1.9 ± 2.0 (92)	0.95 ± 3.1 (42)	8.8 ± 1.6 (39)
Partially aerated (547)	11.4 ± 1.8 (451)	17.0 ± 2.0 (456)	4.9 ± 3.0* (335)	2.0 ± 2.3 (114)	1.7 ± 2.5* (70)	13.5 ± 1.49* (34)
Rock filter (169)	9.3 ± 1.7*,** (168)	17.1 ± 1.8 (165)	3.4 ± 2.2** (151)	–	–	–
Sand filter (122)	7.7 ± 2.1*,** (119)	13.2 ± 2.1*,** (114)	2.3 ± 2.9** (108)	–	–	–
Cover (16)	9.3 ± 1.6 (13)	11.9 ± 1.8 (13)	–	–	–	–
Wetland (15)	7.2 ± 2.1 (12)	11 ± 1.9 (14)	3.7 ± 3.4 (11)	–	–	–
MBBR (22)	9.7 ± 1.9 (19)	13.9 ± 1.9 (22)	0.98 ± 2.3*,** (21)	–	–	–
AGF (47)	3.9 ± 1.9*,** (36)	4.4 ± 1.8*,** (40)	0.66 ± 2.7*,** (35)	–	–	–
CFF (53)	4.1 ± 1.7*,** (46)	5.2 ± 1.8*,** (48)	0.63 ± 2.6*,** (45)	–	–	–
Activated sludge (183)	5.8 ± 2.1*,** (158)	6.8 ± 2.3*,** (159)	1.27 ± 3.6*,** (133)	1.6 ± 2.8 (61)	6.3 ± 2.4*,** (42)	10.7 ± 1.9 (35)
Chemical precipitation (14)	–	–	–	0.74 ± 2.3*,** (14)	–	–

* Significant difference with ‘facultative’ group (*α* = 0.05).

** Significant difference with ‘partially aerated’ group (*α* = 0.05).

**Table 2 ∣:** Summary of multiple variable regression (natural log transformed NH_3_ effluent concentrations)

Predictor variables	$\hat{\beta }$	95% confidence intervals	Effect description
Intercept (technology = basic)	0.94*	0.81 to 1.07	NH_3_ = 2.56 mg/L at median T and NLR
Technology = advanced	−1.61*	−1.86 to −1.39	80% reduction in NH_3_ (when T and NLR at median) compared with basic
Technology = mid-level	−0.011	−0.18 to 0.16	Nonsignificant effect in NH_3_
Technology = aeration	0.309*	0.13 to 0.48	36% increase in NH_3_ (when T and NLR at median) compared with basic
Average annual temperature (linear term)^a^	−0.0527*	−0.072 to 0.033	Nonlinear quadratic effect on NH_3_. Lower temperatures increase NH_3_ until inflection point at 16 °C, where higher temperatures increase NH_3_
Average annual temperature^b^ (squared term)^a^	0.0071*	0.0040 to 0.010	
Nitrogen surface loading rate^b^	0.101*	0.075 to 0.13	For each unit of NLR, NH_3_ increases by 10.6%

a Centered around median (12.4 °C).

b Centered around median (1.81 kg N/acre-day).

* Significance code: 0.001.

## Data Availability

Data cannot be made publicly available; readers should contact the corresponding author for details.

## HIGHLIGHTS

- Analyzed the performance of 1,412 wastewater treatment lagoons (WTLs).
- Facultative and partially aerated WTLs had elevated average effluent concentrations for organics and nutrients.
- Facultative WTLs had higher average NH_3_ concentrations correlated with cold temperatures and nitrogen loading rate.
- Upgraded WTLs, such as moving bed-biofilm reactors, aerated gravel filters, and complete mix ponds, had the lowest NH_3_ effluent concentrations.
