# Supplementary material for: Evaluation of nutrients in effluent from wastewater treatment lagoons discharging to surface water in the United States
Source: Water Sci Technol. Author manuscript; Available in PMC 2026 Sep 17. (PMC13583163; doi:10.2166/wst.2026.261)
Supplement: Supplementary file 1 [file NIHMS2194705-supplement-Supplementary_Information.docx]

**SUPPLEMENTARY MATERIAL**

**Table S1 |** Cutoff ranges for removed monthly effluent concentrations

| **Parameter** | **Min and Max Values (mg/L)** |
| --- | --- |
| BOD/cBOD | 0 – 200 |
| TSS | 0 – 200 |
| NH_3_ | 0 – 80 |
| TP | 0 – 20 |
| NO_3_/NO_2_ | 0 – 40 |
| TN | 0 – 100 |

**Figure S1 |** Distribution of facility-level average effluent concentrations for BOD (top left), TSS (top right), NH_3_ (middle left), TP (middle right), TN (bottom left), and NO_3_ (bottom right). All distributions were right skewed and therefore natural log transformed for analysis.

**Table S2 |** Significant Tukey–Kramer technology pairs for NH_3_

|  | **Log transformed** | | | **Back transformed (geometric mean)** | | |  |
| --- | --- | --- | --- | --- | --- | --- | --- |
| **Technology pair** | **Difference** | **Lower CI** | **Upper CI** | **Ratio of geometric means** | **Lower CI** | **Upper CI** | **Adjusted *p*-value** |
| Aerated-Activated Sludge | 1.34 | 0.99 | 1.69 | 3.81 | 2.68 | 5.43 | 0.00 |
| AGF-Activated Sludge | –0.66 | –1.31 | 0.00 | 0.52 | 0.27 | 1.00 | 0.05 |
| Facultative-Activated Sludge | 0.66 | 0.30 | 1.03 | 1.94 | 1.35 | 2.79 | 0.00 |
| Rock Filters-Activated Sludge | 0.96 | 0.55 | 1.37 | 2.61 | 1.73 | 3.93 | 0.00 |
| Sand Filters-Activated Sludge | 0.59 | 0.14 | 1.04 | 1.81 | 1.16 | 2.83 | 0.00 |
| AGF-Aerated | –2.00 | –2.61 | –1.38 | 0.14 | 0.07 | 0.25 | 0.00 |
| CFF-Aerated | –1.91 | –2.45 | –1.38 | 0.15 | 0.09 | 0.25 | 0.00 |
| Facultative-Aerated | –0.68 | –0.96 | –0.40 | 0.51 | 0.38 | 0.67 | 0.00 |
| MBBR-Aerated | –1.61 | –2.39 | –0.83 | 0.20 | 0.09 | 0.43 | 0.00 |
| Rock Filters-Aerated | –0.38 | –0.72 | –0.04 | 0.68 | 0.49 | 0.96 | 0.01 |
| Sand Filters-Aerated | –0.75 | –1.13 | –0.37 | 0.47 | 0.32 | 0.69 | 0.00 |
| Covers-AGF | 1.83 | 0.54 | 3.12 | 6.22 | 1.71 | 22.58 | 0.00 |
| Facultative-AGF | 1.32 | 0.70 | 1.94 | 3.74 | 2.01 | 6.95 | 0.00 |
| Rock Filters-AGF | 1.62 | 0.97 | 2.26 | 5.03 | 2.63 | 9.61 | 0.00 |
| Sand Filters-AGF | 1.25 | 0.58 | 1.92 | 3.49 | 1.78 | 6.82 | 0.00 |
| Wetlands-AGF | 1.72 | 0.53 | 2.92 | 5.61 | 1.70 | 18.48 | 0.00 |
| Covers-CFF | 1.75 | 0.49 | 3.00 | 5.73 | 1.63 | 20.11 | 0.00 |
| Facultative-CFF | 1.24 | 0.69 | 1.78 | 3.45 | 2.00 | 5.95 | 0.00 |
| Rock Filters-CFF | 1.53 | 0.96 | 2.11 | 4.64 | 2.61 | 8.25 | 0.00 |
| Sand Filters-CFF | 1.17 | 0.56 | 1.77 | 3.21 | 1.76 | 5.87 | 0.00 |
| Wetlands-CFF | 1.64 | 0.49 | 2.80 | 5.17 | 1.63 | 16.42 | 0.00 |
| MBBR-Covers | –1.44 | –2.82 | –0.07 | 0.24 | 0.06 | 0.94 | 0.03 |
| MBBR-Facultative | –0.93 | –1.71 | –0.15 | 0.39 | 0.18 | 0.86 | 0.01 |
| Rock Filters-MBBR | 1.23 | 0.42 | 2.03 | 3.42 | 1.53 | 7.63 | 0.00 |
| Sand Filters-MBBR | 0.86 | 0.04 | 1.68 | 2.37 | 1.04 | 5.39 | 0.03 |
| Wetlands-MBBR | 1.34 | 0.05 | 2.62 | 3.81 | 1.05 | 13.76 | 0.03 |

**Table S3 |** Significant Tukey–Kramer technology pairs for BOD

|  | **Log transformed** | | | **Back transformed (geometric mean)** | | |  |
| --- | --- | --- | --- | --- | --- | --- | --- |
| **Technology pair** | **Difference** | **Lower CI** | **Upper CI** | **Ratio of geometric means** | **Lower CI** | **Upper CI** | **Adjusted *p*-value** |
| Aerated-Activated Sludge | 0.67 | 0.48 | 0.85 | 1.95 | 1.62 | 2.35 | 0.00 |
| AGF-Activated Sludge | –0.41 | –0.78 | –0.04 | 0.66 | 0.46 | 0.96 | 0.02 |
| CFF-Activated Sludge | –0.35 | –0.68 | –0.02 | 0.70 | 0.50 | 0.98 | 0.03 |
| Facultative-Activated Sludge | 0.66 | 0.47 | 0.84 | 1.93 | 1.60 | 2.32 | 0.00 |
| MBBR-Activated Sludge | 0.51 | 0.03 | 0.99 | 1.66 | 1.03 | 2.70 | 0.03 |
| Rock Filters-Activated Sludge | 0.46 | 0.24 | 0.68 | 1.59 | 1.27 | 1.98 | 0.00 |
| Sand Filters-Activated Sludge | 0.28 | 0.03 | 0.52 | 1.32 | 1.03 | 1.68 | 0.01 |
| AGF-Aerated | –1.08 | –1.42 | –0.73 | 0.34 | 0.24 | 0.48 | 0.00 |
| CFF-Aerated | –1.02 | –1.33 | –0.71 | 0.36 | 0.27 | 0.49 | 0.00 |
| Rock Filters-Aerated | –0.21 | –0.39 | –0.03 | 0.81 | 0.68 | 0.97 | 0.01 |
| Sand Filters-Aerated | –0.39 | –0.60 | –0.19 | 0.68 | 0.55 | 0.83 | 0.00 |
| Covers-AGF | 0.87 | 0.23 | 1.52 | 2.39 | 1.26 | 4.56 | 0.00 |
| Facultative-AGF | 1.07 | 0.72 | 1.41 | 2.90 | 2.05 | 4.11 | 0.00 |
| MBBR-AGF | 0.92 | 0.35 | 1.48 | 2.51 | 1.43 | 4.41 | 0.00 |
| Rock Filters-AGF | 0.87 | 0.51 | 1.24 | 2.39 | 1.66 | 3.45 | 0.00 |
| Sand Filters-AGF | 0.69 | 0.31 | 1.06 | 1.99 | 1.36 | 2.90 | 0.00 |
| Covers-CFF | 0.81 | 0.19 | 1.44 | 2.25 | 1.21 | 4.21 | 0.00 |
| Facultative-CFF | 1.01 | 0.70 | 1.32 | 2.73 | 2.00 | 3.73 | 0.00 |
| MBBR-CFF | 0.86 | 0.32 | 1.40 | 2.36 | 1.37 | 4.06 | 0.00 |
| Rock Filters-CFF | 0.81 | 0.48 | 1.14 | 2.25 | 1.62 | 3.14 | 0.00 |
| Sand Filters-CFF | 0.63 | 0.28 | 0.97 | 1.87 | 1.32 | 2.64 | 0.00 |
| Rock Filters-Facultative | –0.19 | –0.38 | –0.01 | 0.82 | 0.68 | 0.99 | 0.03 |
| Sand Filters-Facultative | –0.38 | –0.59 | –0.17 | 0.68 | 0.56 | 0.84 | 0.00 |

**Table S4 |** Significant Tukey–Kramer technology pairs for TSS

|  | **Log transformed** | | | **Back-transformed (geometric mean)** | | |  |
| --- | --- | --- | --- | --- | --- | --- | --- |
| **Technology pair** | **Difference** | **Lower CI** | **Upper CI** | **Ratio of geometric means** | **Lower CI** | **Upper CI** | **Adjusted p-value** |
| Aerated-Activated Sludge | 0.92 | 0.71 | 1.12 | 2.51 | 2.04 | 3.08 | 0.00 |
| AGF-Activated Sludge | –0.43 | –0.82 | –0.03 | 0.65 | 0.44 | 0.97 | 0.02 |
| Facultative-Activated Sludge | 1.05 | 0.83 | 1.26 | 2.85 | 2.30 | 3.51 | 0.00 |
| MBBR-Activated Sludge | 0.72 | 0.16 | 1.28 | 2.06 | 1.18 | 3.59 | 0.00 |
| Rock Filters-Activated Sludge | 0.92 | 0.68 | 1.17 | 2.52 | 1.97 | 3.23 | 0.00 |
| Sand Filters-Activated Sludge | 0.66 | 0.39 | 0.94 | 1.94 | 1.47 | 2.55 | 0.00 |
| AGF-Aerated | –1.35 | –1.72 | –0.98 | 0.26 | 0.18 | 0.38 | 0.00 |
| CFF-Aerated | –1.19 | –1.53 | –0.85 | 0.30 | 0.22 | 0.43 | 0.00 |
| Sand Filters-Aerated | –0.26 | –0.49 | –0.02 | 0.77 | 0.61 | 0.98 | 0.02 |
| Covers-AGF | 0.99 | 0.28 | 1.71 | 2.70 | 1.32 | 5.51 | 0.00 |
| Facultative-AGF | 1.47 | 1.10 | 1.85 | 4.36 | 3.01 | 6.33 | 0.00 |
| MBBR-AGF | 1.15 | 0.51 | 1.78 | 3.15 | 1.67 | 5.95 | 0.00 |
| Rock Filters-AGF | 1.35 | 0.96 | 1.75 | 3.87 | 2.61 | 5.74 | 0.00 |
| Sand Filters-AGF | 1.09 | 0.68 | 1.50 | 2.97 | 1.97 | 4.49 | 0.00 |
| Wetlands-AGF | 0.91 | 0.20 | 1.63 | 2.49 | 1.22 | 5.09 | 0.00 |
| Covers-CFF | 0.84 | 0.14 | 1.54 | 2.31 | 1.15 | 4.65 | 0.01 |
| Facultative-CFF | 1.32 | 0.98 | 1.66 | 3.74 | 2.65 | 5.26 | 0.00 |
| MBBR-CFF | 0.99 | 0.37 | 1.61 | 2.70 | 1.45 | 5.01 | 0.00 |
| Rock Filters-CFF | 1.20 | 0.83 | 1.56 | 3.31 | 2.29 | 4.78 | 0.00 |
| Sand Filters-CFF | 0.93 | 0.55 | 1.32 | 2.55 | 1.73 | 3.74 | 0.00 |
| Wetlands-CFF | 0.76 | 0.06 | 1.46 | 2.13 | 1.06 | 4.29 | 0.02 |
| Sand Filters-Facultative | –0.38 | –0.62 | –0.14 | 0.68 | 0.54 | 0.87 | 0.00 |

**Table S5 |** Significant Tukey–Kramer technology pairs for TP

|  | **Log transformed** | | | **Back-transformed (geometric mean)** | | |  |
| --- | --- | --- | --- | --- | --- | --- | --- |
| **Technology pair** | **Difference** | **Lower CI** | **Upper CI** | **Ratio of geometric means** | **Lower CI** | **Upper CI** | **Adjusted *p*-value** |
| Chemical Precipitation-Activated Sludge | –0.75 | –1.38 | –0.11 | 0.47 | 0.25 | 0.90 | 0.01 |
| Chemical Precipitation-Aerated | –1.01 | –1.62 | –0.40 | 0.36 | 0.20 | 0.67 | 0.00 |
| Facultative-Chemical Precipitation | 0.93 | 0.31 | 1.55 | 2.54 | 1.37 | 4.72 | 0.00 |

**Table S6 |** Significant Tukey–Kramer technology pairs for TN

|  | **Log Transformed** | | | **Back-Transformed (geometric mean)** | | |  |
| --- | --- | --- | --- | --- | --- | --- | --- |
| **Technology Pair** | **Difference** | **Lower CI** | **Upper CI** | **Ratio of geometric means** | **Lower CI** | **Upper CI** | **Adjusted p-value** |
| Facultative-Aerated | –0.44 | –0.72 | –0.16 | 0.65 | 0.49 | 0.86 | 0.00 |

**Table S7 |** Significant Tukey–Kramer technology pairs for NO_3_

|  | **Log transformed** | | | **Back-transformed (geometric mean)** | | |  |
| --- | --- | --- | --- | --- | --- | --- | --- |
| **Technology pair** | **Difference** | **Lower CI** | **Upper CI** | **Ratio of geometric means** | **Lower CI** | **Upper CI** | **Adjusted *p*-value** |
| Aerated-Activated Sludge | –1.30 | –1.75 | –0.84 | 0.27 | 0.17 | 0.43 | 8×10^–10^ |
| Facultative-Activated Sludge | –1.89 | –2.40 | –1.38 | 0.15 | 0.09 | 0.25 | 2×10^–14^ |
| Facultative-Aerated | –0.59 | –1.05 | –0.14 | 0.55 | 0.35 | 0.87 | 6×10^–3^ |

**Figure S2 |** Histogram of average annual temperatures of the identified lagoon facilities.

**Figure S3 |** Histogram of nitrogen surface loading rates for evaluated lagoon facilities. Only the surface loading of the facilities with sufficient ammonia effluent data are shown.

**Figure S4 |** Graphical diagnostic of normality for multiple regression including Q-Q plot (top left), distribution of residuals (top right), fitted values versus residuals (bottom left), and residuals versus index (bottom right)

**Table S8 |** General variable influence factor for multivariate regression

| **Predictor** | **General VIF** |
| --- | --- |
| Temperature | 1.08 |
| Temperature^2^ | 1.09 |
| Nitrogen surface loading | 1.10 |
| Technology | 1.21 |
